# A Chatbot to Meet Parents’ Information Needs for Sickle Cell Trait Newborn Screening Results: Multiple Methods Formative Study

**DOI:** 10.2196/86022

**Published:** 2026-04-30

**Authors:** Anne C Madeo, Courtney Gauchel, Emerson Borsato, Amy Gaviglio, Kimberly A Kaphingst, Guilherme Del Fiol, Karen Eilbeck

**Affiliations:** 1 Department of Population Health Sciences, University of Utah, Salt Lake City, UT, United States; 2 Department of Biomedical Informatics, University of Utah, 421 Wakara Way, Salt Lake City, UT, 84108, United States, 1 801 5814080; 3 Connetics Consulting LLC, Minneapolis, MN, United States; 4 Department of Communication, University of Utah, Salt Lake City, UT, United States

**Keywords:** sickle cell trait, patient care team, neonatal screening, public health, parents, infant, newborn, digital health, conversational agent, chatbot

## Abstract

**Background:**

Newborn screening (NBS), a mandated public health intervention, allows the identification of babies with potentially life-threatening disorders and facilitates disease diagnosis and management before the onset of symptoms. While NBS saves lives, the process can be fraught with anxiety and unanswered questions from parents or guardians of newborns, especially as they wait for an appointment with a clinician.

**Objective:**

This study aimed to describe the development and testing of an educational chatbot (NBSchat) to address the emotional support and information needs of parents of newborns identified with sickle cell trait via NBS.

**Methods:**

NBSchat, a fully scripted (ie, rule-based) chatbot, was developed by a multidisciplinary team and evaluated through a sequential multiple methods study, including interviews and a survey. To inform chatbot design, we conducted semistructured interviews with 11 adults—5 clinicians who work with parents of infants identified with sickle cell trait through NBS and 6 parents of infants aged 12 months or less—using the critical incident technique and think-aloud tasks while using a prototype of NBSchat. Transcripts underwent thematic analysis. In a survey, 250 parents of infants aged 12 months or less without abnormal NBS results were shown a mock newborn screening result letter and then interacted with NBSchat, after which they self-reported emotional and attitudinal outcomes before and after the simulated exposure.

**Results:**

Feedback from interviews confirmed that parents are distressed by trait results and actively seek information and reassurance. Thematic analysis indicated that NBSchat provided reliable, accurate information that parents wanted and had the potential to reduce negative emotions (eg, provide relief and reduce stress). Key strengths included addressing an immediate health concern and offering reassurance. The results of the postintervention survey indicated that, compared to pre-exposure scores, participants reported significantly lower negative emotions (mean 7.0 [SD 3.2] vs 5.8 [SD 3.2] out of 12; mean difference −1.2, 95% CI −1.57 to −0.83; *P*<.001), improved positive emotions (reflected by a decrease in the reverse-coded positive emotion score; mean 8.6 [SD 4.2] vs 7.8 [SD 4] out of 16; mean difference −0.8, 95% CI −1.27 to −0.37; *P*<.001), and reduced uncertainty (mean 6.5 [SD 3] vs 5.5 [SD 3.4] out of 12; mean difference −1, 95% CI −1.42 to −0.58; *P*<.001). Parents noted that NBSchat provided immediate reassurance and was convenient to access. They further reported that the predefined, structured questions in the script helped guide their learning and understanding.

**Conclusions:**

Overall, participants who interacted with NBSchat found it to be acceptable, with improved emotional measures after its use. Future research will investigate the outcomes of using the chatbot and its implementation in a pragmatic randomized controlled trial.

## Introduction

Newborn screening (NBS) identifies serious, treatable conditions in infants before symptoms appear, enabling timely intervention and improved outcomes. In the United States, NBS is administered by state public health programs [1] and is routinely performed within the first 24 to 48 hours of life, unless parents opt out [2]. Most results are normal and reported to the birth hospital or pediatrician, who informs families only if follow-up is needed. For urgent conditions, the laboratory calls the health care provider directly to expedite care. Nonurgent findings, such as sickle cell trait (SCT), are often mailed to both the parents and the infant’s clinician, although processes vary by state [3]. Trait status means the infant is healthy but carries one altered gene that can be passed to future children. Health departments commonly use follow-up teams to communicate results and direct families to next steps, which typically involve the primary care provider, a genetic counselor, or a specialist.

Adverse psychosocial outcomes among parents receiving positive NBS results have long been recognized, particularly in conditions such as cystic fibrosis and sickle cell disease. Early studies highlighted anxiety linked to poor understanding of NBS results [4], and a 2004 systematic review of prenatal and newborn screening underscored this concern. More recent work shows that parents of infants with positive NBS results have higher odds of depression (OR 6.06) and stress (OR 3.2) [5]. Prior research indicates that while the initial shock of the unexpected genetic information is unavoidable, other factors that contribute to distress can be addressed through education and improved communication. These factors include filling knowledge gaps about the condition, genetics, and the next medical steps. For example, autosomal disease inheritance patterns are often confusing concepts to parents [6,7] and can be addressed by education and counseling [8,9]. A 2020 systematic review of 92 studies concluded that unexpected NBS and unsolicited genetic findings continue to cause significant distress, but that risks can be reduced through comprehensive, multimodal parent education tailored to timing and preferences [10]. Quantitative studies similarly show that well-informed parents experience less long-term anxiety, yet the uptake of genetic counseling, which has been shown to reduce guilt and anxiety, remains low, with only one-third of eligible families receiving it [11]. Parents frequently struggle to meet their informational and emotional needs as they move through the process from screening to confirmatory testing and new clinical care. Meanwhile, limited genetic counseling capacity creates a bottleneck [12,13], and state NBS follow-up teams often spend substantial time addressing parental anxiety about trait findings, such as SCT, before families connect with primary care providers or genetic counselors [14]. Trait results for hemoglobinopathies such as SCT constitute the largest portion of returned NBS results [15,16]. Both recent and historical literature emphasize that genetic counseling is central to returning SCT results, as it reduces distress, clarifies that SCT is not a disease, and provides essential education about inheritance and reproductive implications [14,17,18]. Access to genetic counseling services can be uneven due to workforce shortages [19] and regional variation in genetic counselor concentration [20].

Digital health tools, such as conversational agents (“chatbots”), have the potential to address challenges in the delivery of NBS results efficiently and at scale and provide consistency across content shared [21]. Chatbots are computer programs that simulate human conversations by using predetermined rule–based responses or artificial intelligence algorithms. Their use in health care has been increasing and includes reminding patients to take medications [22], offering genetic testing [23], and providing brief mental health therapy [24]. User evaluation of digital health interventions, at an early stage of the development process, is essential to ensure that the resulting tool is acceptable and useful to the target population. Chatbots have not previously been used to educate parents about NBS or SCT. This education is typically provided through handouts or in-person instruction. Incorporating a chatbot into a state’s NBS workflow may improve knowledge among individuals who are less likely to accept or have access to genetic counseling. The objectives of this study were to (1) describe the development of a chatbot (NBSchat) that provides information and emotional support to parents who receive an NBS screen-positive result for SCT, (2) explore users’ and clinicians’ experiences and seek feedback to help guide and refine the design of NBSchat content and usability, and (3) compare parent participants’ emotional and cognitive outcomes before and after using NBSchat.

## Methods

### Study Design

Overall, this was an exploratory sequential multiple methods study consisting of semistructured interviews using the critical incident method and a quantitative survey where participants were asked to rate emotional and cognitive outcomes before and after using NBSchat.

### Ethical Considerations

The study was reviewed by the University of Utah Institutional Review Board (IRB), which deemed it exempt (IRB_00179621). Interview participants were provided with a concise study summary with a consent cover letter prior to their interviews and were compensated for taking part with US $50 via a secure online funds transfer system. Verbal consent was obtained at the beginning of each interview. Participants were informed that a transcript of their audio recording would be prepared and deidentified for the study. In accordance with the institutional review board protocol, participants were also informed that the audio files and transcripts would be destroyed after the study. Survey participants were provided with a consent cover letter prior to beginning the survey, and those who completed the survey were compensated with US $14.75 for their participation in accordance with the policies of the survey panel provider. Participants were informed that their responses would be kept confidential, that no identifying information would be disclosed, and that results would be reported in aggregate. Chatbot logs contain timestamps and paths through the conversation for each user; they do not contain identifying information.

### Initial NBSchat Design

To ensure that we developed a robust, patient-focused technology that is tailored to parents’ needs, we followed a participatory approach. Guided by principles of health communication [25], an interdisciplinary team that included genetic counselors, informaticists, a labor and delivery nurse, an NBS expert, and a communication scientist developed an initial draft of the chatbot script [26]. In the initial draft, we sought to address information gaps that cause preventable anxiety: not understanding the implications of the result, wondering about the status of other family members and reproductive health, and navigating the next steps in the health care process. The script was then iteratively refined based on the group’s feedback through meetings and offline discussions.

**Figure 1.**
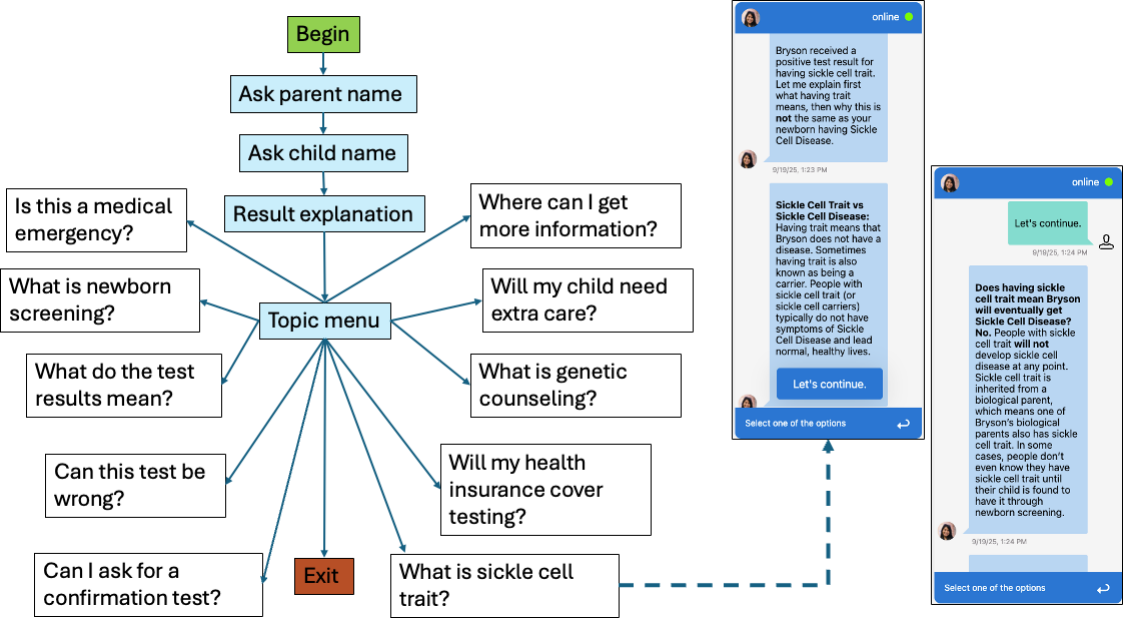
The flow of NBSchat. All patients navigate to the topic menu, after which the topics are optional. The example conversation shown addresses the question, “What is sickle cell trait?” The screenshots shown are simulated example screens for demonstration purposes only and do not depict a real patient or actual participant conversation.

NBSchat was developed using the GARDE-Chat [27] platform developed by investigators at the University of Utah. GARDE-Chat includes a chatbot authoring tool that enables the creation and management of multiple rule-based, large language model (LLM)–based, or hybrid chatbots. LLM-based chatbots, such as ChatGPT, are known to occasionally provide inaccurate information (also known as “hallucinations” [28]) and answer questions that fall outside the predefined boundaries of a chatbot. To avoid these issues, we chose to use a rule-based approach with a predefined conversation script and a fixed set of possible questions and responses that provide accurate and consistent information to parents (Figure 1).

### Interviews

#### Study Participants

To evaluate the content and flow of NBSchat, we conducted in-depth interviews via Zoom from December 10, 2024, to February 7, 2025 [29]. During the study timeframe, we were unable to recruit additional parents of children with SCT. As a result, participants included 6 adult parents of children less than 12 months old (1 parent of a newborn identified with SCT through NBS and 5 parents of newborns who had not received a positive NBS result) and 5 genetic counselors who speak with parents whose newborns were identified as having SCT through NBS.

Participants were recruited through various means. Clinicians were recruited through emails sent to members of the National Society of Genetic Counselors who indicated that their specialization included “Public Health/Newborn Screening.” Parents of newborns were recruited through the University of Utah Office of Research Participant Advocacy. In addition, parents of newborns with SCT were identified through emails seeking partnerships with state chapters of the Sickle Cell Disease Association of America, postings on social media pages, and advertisements at clinics that provide care to infants with SCT. Participants contacted the team via a project-specific email address and were screened via a set of 9 questions to confirm they met eligibility criteria. We invited participants to refer individuals who they thought would be eligible and interested in participating.

#### Interview Methods

Interview guides were tailored to each eligibility group and used the critical incident technique [30] and a think-aloud task [31]. The critical incident technique was selected to allow the participants to be as specific as possible and provide rich data in their own words [30,32]. For parents of an infant identified with SCT, the critical incident was defined as the moment they first received their child’s NBS result. Parents of a newborn who did not receive a positive screen for any condition were provided an anonymized positive newborn screen letter and asked to imagine that they had received the letter 2 weeks after their infant was born. Clinicians were asked to describe a specific instance when they discussed with a parent about their child’s SCT NBS screen-positive result.

To assess the usability of NBSchat, after describing the critical incident, each participant was asked to load the chatbot on the device they used to connect to the interview, share their screen, and describe their thoughts and reactions (“think-aloud”) while they navigated NBSchat and looked for information that they thought would be important to a parent of a newborn recently identified with SCT. After a participant completed this think-aloud task, interviewers probed to ascertain issues pertaining to NBSchat’s content and navigation. We chose not to undertake iterative changes to the chatbot design during the interviews so that we could assess whether certain elements of NBSchat were more or less appealing to both groups of participants. At the close of the interview, all participants were asked sociodemographic questions.

### Interview Data Analysis

All interviews were audio-recorded and transcribed verbatim by a professional transcription service. We aimed to produce actionable, accurate insights that could be incorporated into the design of NBSchat; therefore, qualitative data collected through the interviews were analyzed using the Rapid and Rigorous Qualitative Data Analysis technique [33,34]. The research team developed a phase 1 matrix in Microsoft Excel that reflected the topics assessed in the interview guide and their preliminary codes, interpreting the data [35]. All members of the research team read the phase 1 matrix and discussed the goals of the analysis. Based on these discussions, authors CG and ACM further reduced the data in the phase 1 matrix and created a phase 2 matrix that provided actionable feedback with supporting quotes addressing the overarching research question. The phase 2 matrix incorporated analysts’ “focused codes [36].” Data reduction and condensation occurred iteratively until the team agreed that the final phase table addressed our overarching research question. Disagreements were resolved through discussion.

### Survey

#### Participants

The survey was deployed by Prodege to a sample of 330 participants, who were similar to the United States’ demographics in terms of race, ethnicity, and education level. Eligibility criteria included being 18 years or older, being the parent of a child 12 months old or younger, and not having a child identified with a health condition through NBS. The survey was conducted from March 13, 2025, to May 1, 2025. Of the 277 participants who completed the survey, 250 passed the attention checks and successfully launched NBSchat.

#### Measures

##### Demographics

Demographic data, including accessibility to and use of mobile technology [37], were collected.

##### Health Literacy and Genetic Knowledge

The survey assessed participants’ understanding of genetic test terminology using items adapted from *Measuring Genetic Knowledge: A Brief Survey Instrument for Adolescents and Adults* [38]. This is a 7-question instrument using true or false items. We utilized 6 of the 7 items from this instrument. In addition, we assessed participants’ general health literacy using the BRIEF Health Literacy Screening Tool [39,40]. This is a 4-question survey measured using a 5-point Likert scale with a range of 4 to 20.

##### Self-Efficacy and Emotional Responses

Communication self-efficacy was assessed using the Ask, Understand, Remember (AURA) survey [41], a 4-item measure administered on a 4-point Likert scale. Items were adapted to be about the child’s case. Psychosocial distress was assessed with 10 items from 3 subscales (negative feelings, positive feelings, and uncertainty) derived from the Feelings About Genomic Testing Results (FACToR). All items were measured on a 5-point Likert scale [42]. These measures were assessed both before and after using NBSchat. The positive emotion items were reverse-coded, so lower scores corresponded to reduced negative affect and greater positive emotion. Items were adapted from the original to reflect the screening result.

##### Usability and Usefulness

NBSchat usability was assessed using the System Usability Scale (SUS) [43]*,* a 10-item questionnaire with a 5-point Likert scale, where scores are converted to a 0 to 100 usability score. Items were adapted to reflect the software being a chatbot, and open-ended comments were also collected.

### Procedure

A survey instrument was developed using the Qualtrics platform and incorporated 3 attention check items to assess response quality [44-46]. Participants who failed one or more attention checks were removed from the final analysis to minimize the impact of insincere survey takers. Analyses were conducted using complete cases; sample sizes varied slightly by analysis due to item nonresponse. Participant knowledge of genetics and health literacy was assessed; then, participants were presented with a simulated NBS result indicating that a newborn had screened positive for SCT and were asked to complete a modified version of the AURA and FACToR instruments to capture baseline self-efficacy and emotional responses. Following this, participants interacted with NBSchat and were then asked to complete the AURA and FACToR questions to evaluate changes in outcomes. Participants then provided usability and usefulness feedback, followed by demographic information. Given the exploratory mixed methods design, recruitment targets were inflated to account for an anticipated incomplete rate of approximately 30%, with the goal of obtaining at least 100 open-ended responses to support robust and diverse qualitative perspectives and a final analytic sample sufficient to detect small pre-post changes in AURA and the FACToR subscale scores. Missing data were minimal (1 of 250 participants missed one item on a multi-item scale;<0.5%). Scale scores were computed as the mean of completed items. Given the negligible level of missingness, no additional imputation procedures were conducted.

### Survey Data Analysis

Survey data were analyzed using Microsoft Excel. Descriptive data, including means and standard deviations, were obtained for all measures. The significance of differences in measures before and after using NBSchat was determined using the paired *t* test, with statistical significance assessed as a *P*<.05. Open-ended comments were coded into 3 categories: positive comment, negative comment, and neutral comment during a content analysis. After reading all the comments, 2 reviewers agreed upon codes and selected representative quotes. The integration of survey with interview data occurred by comparing themes identified in interviews with patterns observed in survey responses.

## Results

### Interviews

#### Overview

Analysis of interview transcripts revealed 4 overarching categories of themes that capture parents’ experiences and needs following an NBS screen-positive result for SCT (carrier status). First, parents rely on multiple sources of information, including online searches, health care providers, phone hotlines, and personal networks. Second, parents aimed to reduce negative emotions and clarify immediate health concerns via information seeking. Third, parents and clinicians felt that NBSchat may help reduce negative emotions and address immediate health concerns, emphasizing its potential to reduce worry, provide actionable information, and offer reassurance. Finally, NBSchat was described as a tool that could bridge structural and communication gaps in care—including language barriers, geographic distance to clinics, long wait times for appointments, and challenges related to health literacy. Interviews were conducted until thematic saturation was reached, defined as the point at which no new themes emerged in subsequent interviews. Together, these themes highlight the barriers parents encounter when navigating the NBS system and the opportunities for digital tools to provide timely, understandable, and supportive information. Exemplar quotes for each theme identified in the interviews are collated in Table S1 (Multimedia Appendix 1) and are described below.

#### Theme 1. Parents Relied on Multiple Sources to Meet Their NBS Information Needs

A discussion of the sources of information parents currently use when navigating the NBS landscape revealed 4 key codes. Health care providers and parents indicated that the internet was a popular avenue for parents trying to understand the results. Clinical providers, such as the child’s pediatrician, are a valuable resource for parents; however, there can be a disconnect between the state NBS follow-up team and the pediatrician’s office regarding the interpretation of results and the transfer of educational materials. For the parents’ interviews, after being shown a standard trait result letter, many participants would call the public health lab for the clarification of the result’s meaning. Finally, parents rely on a network of family and friends whom they also turn to for support.

*I know we shouldn’t Google, but then I would maybe just look over, see what exactly the baby had.*

[Parent-007]

*…the parent received that letter at home, which had our number, and then they would call us there saying, “What does this mean?”*

[Clinician-006]

*If I had a friend or family member who had a baby with a genetic thing pop up, I would reach out to them.*

[Parent-005]

#### Theme 2. Parents Aimed to Reduce Negative Emotions and Clarify Immediate Health Concerns

When parents received SCT results, 2 major themes consistently emerged: parents looked for reassurance to reduce negative emotions and clarification of immediate health concerns. Clinicians described how initial fear and confusion often gave way to relief once parents were reassured that SCT is not a disease and does not pose a long-term health risk. Parents expressed gratitude when told directly that their baby was healthy, and clinicians observed that this reassurance helped alleviate distress. At the same time, parents and clinicians reported a strong desire for practical information to address immediate health concerns. Questions such as whether SCT could make a child sick, what symptoms to watch for, whether diet or treatment changes were necessary, and how SCT differed from sickle cell disease emerged. These findings underscore that both emotional support and clear, actionable guidance are essential for parents to understand and cope with SCT results.

*Whenever I say that [results are most relevant for parents’ future family-planning], that’s when the relief comes out. I would say that people are reassured by the information that’s given.*

[Clinician-004]

*…it [NBS trait results] kind of freaked the parents out and they were like, “Oh, my gosh. What’s going on with our child?” Whereas when they came into the office and we actually sat down and looked at it, like baby’s fine.*

[Clinician-005]

*I think that the first thing I would want to know is, what is sickle cell trait?*

[P-007, parent of infant without NBS experience]

#### Theme 3. Parents and Clinicians Felt That NBSchat May Help Reduce Negative Emotions and Address Immediate Health Concerns

After using the NBSchat prototype in the interview setting, both clinicians and parents felt that it would reduce negative emotions and help address immediate health concerns. One of the parents discussed the stress of not being able to reach the right person when calling a clinician, a situation in which a chatbot could provide immediate assistance. The parents appreciated the immediate feedback indicating that their child did not have the disease, and their questions could be quickly addressed. The menu of questions was seen positively as it provides a pathway to obtain new information.

*Sometimes it’s frustrating also to call phone numbers, especially in this situation, because you don’t go, sometimes, immediately to the person that you wanna talk to...Then it will be stressful. I already have so much going on.*

[Parent-007]

*I think it [additional information] does [provide reassurance]. Even the families where they’re very distressed.*

[Clinician-004]

*I liked that it told me right off the bat the results and everything and that it’s not an emergency and you should talk to your doctor about it. It gave you instructions.*

[Parent-001]

#### Theme 4. Bridge Structural and Communication Gaps in Care

Clinicians and parents agree that language is a prominent issue when relaying trait results. Genetic counselors are concerned about leaving nonnative English speakers confused. Issues with both rural considerations and wait times for a genetic counselor appointment were brought up by clinicians, which highlights the dearth of qualified providers in some parts of the country. Ultimately, the chatbot was viewed as a bridge to parents with limited health literacy. Again, the menu was positively perceived by those with low health literacy to provide a framework to structure information and allow choice in what information to receive.

*I’m glad that it was a menu ‘cause then you guide me on how the terminology goes, and then you give me exactly what I ask for rather than me trying to tell you what I’m asking.*

[Parent-005]

*Then about I’d say probably five, six months is when the patients actually will be scheduled, so they’ve got a very long wait time.*

[Clinician-005]

*I’d say the vast majority prefer or request the phone genetic counseling and that is one reason for that could be that our state is quite rural and the genetic counselors that do this are only located in one city in the state. To physically come here is often very far.*

[Clinician-002]

*[F]or a result like sickle cell trait, I think getting the information across in their language is maybe the most challenging part to make sure that makes sense.*

[Clinician-002]

### Survey

#### Demographic Characteristics

Participant demographics are outlined in Table 1. The majority of participants were White (n=179, 71.6%), women (n=178, 71.2%), and non-Hispanic (n=214, 85.6%). Educational attainment was equally distributed across high school (n=77), associate’s (n=88), and college levels (n=77, 30.8%‐35.2%), with junior high represented at 3.2% (n=8). The majority of respondents reported internet availability at home (n=233, 93.2%), with cellphone ownership at 99.6% (n=249), and all cellphones were reported as smartphones.

**Table 1.** Survey population characteristics (N=250).

Characteristic	Participants, n (%)
Race	
American Indian or Alaska Native	4 (1.6)
Asian or Asian American	14 (5.6)
Black or African American	34 (13.6)
Native Hawaiian or Pacific Islander	1 (0.4)
White	179 (71.6)
Some other ancestry or origin	6 (2.4)
Mixed ancestry	12 (4.8)
Hispanic, Latino, or Spanish origin	
Yes	36 (14.4)
No	214 (85.6)
Gender	
Woman	178 (71.2)
Man	72 (28.8)
Some other way	0 (0)
Education level	
Junior high	8 (3.2)
High school	77 (30.8)
Some college or associate's degree	88 (35.2)
College	77 (30.8)
Current subscription to an internet service at home	
Yes	233 (93.2)
No	17 (6.8)
Type of home internet service	
Dialup	1 (0.4)
High-speed broadband like cable, fiber optic, wireless router, satellite, or digital subscriber line (DSL)	227 (90.8)
Not sure	5 (2)
Blank	17 (6.8)
Cell phone ownership	
Yes	249 (99.6)
No	1 (0.4)
Type of cell phone	
Smartphone	249 (99.6)

#### Health Literacy and Genetic Knowledge

The BRIEF Health Literacy Screening items were measured on a 5-point Likert scale, with a range of 4 to 20, where 20 indicates the highest level of literacy. The sample had a high health literacy average, but a wide range (mean 16.3, SD 3.6). For the genetic knowledge questions, participants correctly answered a mean of 5.72 out of 6 items (SD 0.6; range 0-6). The highest proportion of correct responses (n=241, 96.4%) was for the item “All serious diseases are inherited (false),” while the lowest (n=235, 94%) was for “Healthy parents can have a child with an inherited disease (true).”

#### Self-Efficacy and Emotional Responses

There was no significant difference in communication self-efficacy before versus after using NBSchat (13.5 out of 16 vs 13.4; *P*=.42). FACToR subscales are shown in Table 2. Negative feelings were significantly lower after exposure to NBSchat (7.0 out of 12 vs 5.8; *P*<.001). Positive feelings also improved following exposure to NBSchat, as reflected by a decrease in the reverse-coded positive emotion score (8.6 vs 7.8; *P*<.001). Finally, a small but significant decrease in uncertainty was observed (6.5 vs 5.5; *P*<.001). Statistical significance was set at *α*=.05. As a sensitivity analysis, pre-post comparisons were also conducted using Wilcoxon signed-rank tests, which yielded the same pattern of results and conclusions.

**Table 2.** Feelings About Genomic Testing Results (FACToR) subscales for negative emotions, positive emotions, and uncertainty before and after exposure to NBSchat.

Subscale score	Before NBSchat, mean (SD)	After NBSchat, mean (SD)	Mean difference (95% CI)	Cohen *d*	*P* value
Negative emotions (0‐12)	7 (3.2)	5.8 (3.2)	*−*1.2 (−1.57 to −0.83)	0.3881	<.001
Positive emotions (0‐16)	8.6 (4.2)	7.8 (4)	*−*0.8 (−1.27 to −0.37)	0.2301	<.001
Reduced uncertainty (0‐12)	6.5 (3)	5.5 (3.4)	*−*1 (−1.42 to −0.58)	0.3091	<.001

#### Usability and Usefulness

The mean SUS [43] score was 73 (SD 22), which falls above the commonly accepted threshold of 68, indicating good usability.

Participants addressed the prompt “Please write below what information you found useful in the chatbot.” There were 195 positive comments that described useful attributes of participants’ NBSchat experience. Recurring themes (Table 3) are that participants learned something new, found it reassuring, found it convenient for out-of-hours questions, and appreciated the menu of questions.

There were 30 negative comments that described NBSchat as not useful or listed a problem in its usage. We found instances where NBSchat was not using the correct name for the parent or child, an issue that was raised by participants in some of the interviews. Some participants found the response time too fast, while others found it slow, leading us to implement pauses in the NBSchat that allow the parent to better control the release of information.

**Table 3.** Content analysis of survey participants’ comments about the usefulness of NBSchat.

Theme	Representative quotes
Learned something new	I thought that it was useful how the bot gave an in-depth explanation of the results and what they mean and explaining the difference between sickle cell trait and disease Learning that just because someone has a trait doesn’t mean they have the disease.
Gained reassurance	The reassurance that the trait doesn’t mean Sickle Cell Anemia. …the chat answered my questions and honestly would have made me feel better if this [w]as a real situation.
Convenience	Quickly find out that everything is okay without waiting until the morning, when the doctors’ working day begins. As a worried parent, we would rather have immediate answers rather than waiting for a follow-up with a medical professional.
Menu of questions	I like the predeveloped questions to ask further information. I found it useful that it gave me prequestions to ask further because it could be hard at this moment to even know what to Google and seek out more information.

## Discussion

### Principal Findings

In this study, interviews and surveys with new parents demonstrated a clear information gap surrounding SCT results, which can lead to distress and uncertainty. Parents consistently sought reassurance and rapid answers to immediate health concerns. A key finding of the survey is the significant decrease in both distress (negative emotions) and uncertainty following the use of NBSchat. We also observed a small but significant increase in positive emotions. While some parents were content to find out that SCT is not the same as sickle cell disease, many parents reported taking a deeper dive into the menu of questions, with the most common theme being that they learned something new. The result explanation and the list of further resources were parts of NBSchat that filled an information gap. Overall, the study results suggest that NBSchat is a promising digital health tool for scalable and efficient delivery of NBS information to assist in the return of NBS results.

Because trait results represent the largest category of results returned by NBS programs, follow-up teams frequently receive calls from parents seeking clarification. The problem is compounded by delays: results are often mailed, followed by a lag before discussion with the infant’s clinician and an even longer wait for an appointment with a genetic counselor. The goal of NBSchat is not to replace genetic counseling but to supplement it by preparing parents with foundational knowledge and reducing anxiety so they arrive at appointments more informed [47], more confident, and able to ask targeted, personally relevant questions [48,49]. Given genetic counseling workforce shortages [19] and the limited availability of counselors in many regions [20], NBSchat also represents a practical intermediate solution to address gaps in parent communication and support, reflecting a focus on patient-centered care [50].

Previous research has identified significant variation in SCT disclosure to parents during the newborn period [17,21]. NBSchat offers standardized information to all parents, regardless of where screening was performed, and can be tailored to the unique needs of a state’s NBS program. The clinicians in the study were concerned about the communication of trait results across language barriers; in some cases, even with an interpreter. This is an area where deploying a chatbot in the parents’ first language could be very impactful.

The exploration of usability in the survey found the chatbot to be functional and user-friendly, with only minor areas for potential improvement. According to standard SUS interpretation guidelines, a score of 73 places the tool in the “Good” range and corresponds roughly to a B rating, reflecting positive user experience and acceptance.

The survey found that participants’ communication self-efficacy was high before using NBSchat, and there was no significant change after using it. A mean score of 13.5 out of 16 on the AURA scale represents 84% of the maximum, indicating that participants had strong confidence in asking medical questions, with good perceived understanding and a high likelihood of remembering or using the information effectively. Participants reported feeling confident in engaging in health care discussions and seeking clarification. This likely reflects the sample of survey participants and may not represent the communication self-efficacy of parents who receive a screen-positive result for SCT.

NBSchat can be accessed via a web browser on any internet-connected device, such as a smartphone, tablet, or computer. In 2024, 99% of US adults aged 18 to 49 years reported using the internet [37], and the mean age at first birth in the United States was 27.4 in 2022 [51]; thus, it is likely that the majority of infants in the United States will be born to an adult who uses the internet, whether through a smartphone or other internet-connected device.

The alignment between interview themes and survey findings suggests that user experiences identified in the qualitative phase were not isolated but reflected broader user patterns. The results suggest that NBSchat is an effective and highly scalable approach to delivering patient-tailored education to address the unmet informational needs of parents and alleviate emotional distress. These findings align with previous studies, which provide strong evidence demonstrating the effectiveness of health service delivery and education through scripted chatbots in various health domains, with demonstrated benefits in lowering rates of depression and anxiety [24] and in the delivery of pre-test cancer genetic services and genetic testing [23].

Although recent health care chatbots have started to leverage artificial intelligence–based approaches using LLMs [52], rule-based chatbots are simpler to implement, safer (ie, the design team can ensure that the chatbot only provides accurate, compassionate, and culturally adapted information), constrained to only answer questions about the state’s NBS program and the child’s NBS result, and effective for goal-oriented use cases with a clearly defined end point, such as educating users about NBS results and processes [53]. Participants responded positively to having a menu of options to select for further information, and at least some were unsure what to ask without the structured question format, suggesting that they did not perceive a need for an artificial intelligence–based approach. Furthermore, in prior work using a chatbot to provide information about genetic services and genetic test results, few patients asked open-ended questions, indicating that a rule-based approach may have met the patients’ informational needs [54]. Therefore, scripted chatbots, such as NBSchat, are, at least to this time, an adequate and safer approach for chatbot implementation, especially in sensitive topics such as the explanation of NBS results. This approach also enables each state’s follow-up team to tailor the content unique to their program and update it as necessary through a distributed content authoring approach using the GARDE-Chat platform. Nonetheless, we acknowledge that a hybrid approach, using LLMs at end points in the script, could allow parents to explore more nuanced queries. Future work will explore adapting NBSchat to support free-text questions using a carefully constrained LLM approach, such as a retrieval-augmented generation framework grounded exclusively in authoritative NBS content and explicit scope boundaries to balance flexibility with safety.

Limitations of this study include potential selection bias in the parent interviews and survey toward individuals with higher health literacy and genetic knowledge. Participants in the survey had high genetic knowledge on average, with little variation in scores. We also found that 93.2% (n=233) of the participants had home internet service and 99.6% (n=249) had a smartphone, showing a high level of digital access. Additionally, using attention checks may introduce bias toward higher digital literacy respondents, although we minimized this risk by using plain and unambiguous language. Participants could not be blinded to NBSchat usage, which may have changed their information-seeking behavior because they knew they were being “treated.” The survey relied on self-reported measures, which may be affected by social desirability bias. The simulation of delivering hypothetical test results may not reflect real-world outcomes.

While the survey recruited participants from across the United States and attempted to recruit a representative sample across education levels, the study population reflected relatively high literacy and consistent access to digital technologies. Consequently, participants were not proportionally representative of the ancestral and ethnic groups that bear a disproportionate burden of SCT and disease. Future evaluations of NBSchat should focus on recruitment approaches that better reach these populations, including partnering with newborn screening programs, safety-net health systems, and community-based clinics serving predominantly affected families. Future studies testing NBSchat within these settings and populations are needed to more accurately assess its acceptability and effectiveness among those most affected by SCT. The clinical trial would also demonstrate the generalizability and modularity of NBSchat by adapting it to the particular workflow of a state follow-up team.

The findings should also be interpreted in light of important constraints, including the absence of a control group and the use of hypothetical screening scenarios. Accordingly, the observed pre-post changes in emotional measures cannot be interpreted as causal effects and instead represent preliminary signals supporting the feasibility and potential value of NBSchat, which warrant confirmation in controlled, real-world studies.

In this proof-of-concept demonstration, NBSchat was developed in English. Future iterations of NBSchat are planned to include additional language options, informed by community needs and implementation considerations, to enhance accessibility for linguistically diverse families and ensure the delivery of newborn screening information to all populations.

### Conclusion

We developed and tested NBSchat, a prototype chatbot to support parents who receive an NBS SCT screen-positive result before speaking with a clinician. Both parents and clinicians reported needing to search multiple sources to understand NBS, suggesting that NBSchat could fill this gap by delivering clear, reliable information when results are received. In a simulated evaluation, parents using NBSchat reported reduced negative emotions and greater reassurance, addressing the distress and uncertainty often experienced while awaiting clinical follow-up. These findings represent preliminary signals of potential benefit and support further evaluation of NBSchat in controlled, real-world clinical settings.

## Supplementary material

10.2196/86022 Multimedia Appendix 1 Exemplar quotes for each theme identified in the interviews.

## Abbreviations

AURA: Ask, Understand, Remember
FACToR: Feelings About Genomic Testing Results
LLM: large language model
NBS: newborn screening
SCT: sickle cell trait
SUS: System Usability Scale
