# Supplementary material for: A Chatbot to Meet Parents’ Information Needs for Sickle Cell Trait Newborn Screening Results: Multiple Methods Formative Study
Source: J Med Internet Res. 2026 Apr 30;28:e86022. doi: 10.2196/86022 (PMC13131824; doi:10.2196/86022)
Supplement: Multimedia Appendix 1 [file jmir-v28-e86022-s001.docx]

**Table S1. Exemplar quotes for each theme identified in the interviews.**

| **Parents relied on multiple sources to meet their NBS information needs.** | |
| --- | --- |
| **Code** | **Quote** |
| *Google/internet* | “Some of the information they [parents with a child who had a positive NBS result] probably could get just from Google” C-006, genetic counselor previously employed by a NBS program |
|  | “I know we shouldn't Google, but then I would maybe just look over, see what exactly the baby had” P-007, parent of infant not identified with any health conditions through NBS |
| *Healthcare providers* | “We have information that is sent along with what is hemoglobin disease or hemoglobin traits that is faxed to the provider. I don't think that that's probably ever given out to patients, especially if the provider is not actually saying that the child has a hemoglobin trait, which is what they [parents] are reporting to me...The program here does provide [physicians] with some physical materials. However, we think that there's probably a lack of that actually getting into parent hands.” C-004, clinician who sees parents as part of a state NBS contract |
|  | “The parent letter in [state] seemed pretty good. It's more the letter that—the result cover note that goes to the pediatrician's office—it's a little bit more confusing, and so I think that some pediatricians' offices don't interpret that as well, and so families can come in with some confusion as to is it disease, is it trait, how much do I need to worry about this.” C-001, clinician who sees parents as part of a state NBS contract |
|  | "I guess I'd call my pediatrician after [getting NBS results screen positive letter] 'cause I wouldn't quite know what that meant." P-005, parent of infant without NBS positive result experience |
| *The phone numbers available on the results letter* | "I guess I'd call the phone numbers, next…'cause I wouldn't quite know what that meant." P-005, parent of infant without NBS experience |
|  | “…the parent received that letter at home, which had our number, and then they would call us there saying, ‘What does this mean?’" C-006, clinician who worked for a state NBS program |
|  | “…honestly, my next action would be to try to call the phone number [in the NBS result letter]." P-007, parent of infant without NBS experience |
| *Friends or family* | “I would just tell my husband to see if he maybe gets something more from the letter than I did” P-007, parent of infant without NBS experience |
|  | "If I had a friend or family member who had a baby with a genetic thing pop up, I would reach out to them.” P-005, parent of infant without NBS experience |

| **Parents aimed to reduce negative emotions and clarify immediate health concerns.** | |
| --- | --- |
| **Code** | **Quote** |
| *Reduce negative emotions* | “Whenever I say that [results are most relevant for parents’ future family-planning], that's when the relief comes out. I would say that people are reassured by the information that's given.” C-004, clinician who sees parents as part of a state NBS contract |
|  | “…it [NBS trait results] kind of freaked the parents out and they were like, “Oh, my gosh. What’s going on with our child?” Whereas when they came into the office and we actually sat down and looked at it, like baby’s fine." C-005, clinician who sees parents as part of a state NBS contract |
|  | “The…mom definitely expressed relief when we said that it's—when we gave her reassurance that it's just sickle cell trait, that it's not a long-term health issue, that it's not disease. She expressed that she had been worried and was glad to be able to come in and get that information. " C-001, clinician who sees parents as part of a state NBS contract |
| *Address immediate health concerns* | “what does this result mean and is there anything that's needed reaction wise? ” C-006, clinician who sees parents as part of a state NBS contract |
|  | “very important concern of just could they [infant with SCT NBS results] get sick and what do I need to watch out for” C-006 |
|  | “I think that the first thing I would want to know is, what is sickle cell trait? " P-007, parent of infant without NBS experience |
|  | “…we'll get questions like, ‘do we need to change their diet? Is this tied to anemia? What should they look out for, signs or symptoms? Is there a way to change it?’ I think that they [parents] feel like—it seems like maybe people are looking for an actionable thing to do, either to keep an eye out for something or to change a diet or take a vitamin or something.” C-001, clinician who sees parents as part of a state NBS contract |
|  | “a lot of times for hemoglobinopathies it's, what treatment do they need?” C-004, clinician who sees parents as part of a state NBS contract |

| **Patients and clinicians felt that NBSchat may help reduce negative emotions and address immediate health concerns.** | |
| --- | --- |
| **Code** | **Quote** |
| *Reduce negative emotions* | “’There are over 2.5 million Americans with sickle cell trait.’ That's also good to know, so that you don't feel alone." P-007, parent of infant without NBS experience. |
|  | “Sometimes it's frustrating also to call phone numbers, especially in this situation, because you don't go, sometimes, immediately to the person that you wanna talk to.... Then it will be stressful. I already have so much going on.” P-007 |
|  | “Even though I would still have worries, it [the chatbot] helped ease them a little bit by just telling you it’s not an emergency, just somethin’ you wanna follow up with your doctor about...That was helpful.” P-001, parent of infant without NBS experience |
|  | “I think it [additional information] does [provide reassurance]. Even the families where they're very distressed.” C-004 |
|  | “’Understand this does not mean that your child has sickle cell anemia disease.’ It puts my mind at ease because I don't know how to interpret test results. I don't have that background so that's nice. " P-004, parent of infant without NBS experience |
| *Address immediate health concerns* | “I would probably stop reading [the chatbot] at what do your child's test results mean” P-004, parent of infant without NBS experience |
|  | “I feel I've got what I needed or wanted from this. I have actionable steps." P-004, parent of infant without NBS experience |
|  | "I liked that it told me right off the bat the results and everything and that it’s not an emergency and you should talk to your doctor about it. It gave you instructions." P-001, parent of infant without NBS experience |

| **Bridge structural and communication gaps in care** | |
| --- | --- |
| **Code** | **Quote** |
| *Language* | “I see that confusion more frequently in families that do not speak English as their first language. I think the language barriers can impact how that result is, I guess, heard or understood by the family. I'm sure having twins probably didn't help, but on the contrary, then you could have a specific example where you say, here's the situation for one child and here's the situation for the other. You can see it's different because only one of your kids needs medication. I think the language barrier is the main piece of that. " C-002 |
|  | “…for a result like sickle cell trait, I think getting the information across in their language is maybe the most challenging part to make sure that makes sense. " C-002 |
|  | "Oftentimes, even toward the end of the phone call, I'll get the sense that they don't quite understand what I was trying to explain to them, even though I'm using a translator to assist with communication. Even knowing that there are cultural norms that are different, that I can't account for myself, just because the translation only goes so far.” C-004 |
|  | “I would wonder if there's another language option.” P-005, parent of infant without NBS experience |
| *Rural considerations* | “I'd say the vast majority prefer or request the phone genetic counseling and that is one reason for that could be that our state is quite rural and the genetic counselors that do this are only located in one city in the state. To physically come here is often very far.” C-002 |
| *Wait time for appointment* | “Then about I’d say probably five, six months is when the patients actually will be scheduled, so they’ve got a very long wait time....” C-005 |
| *Low health literacy* | "I'm glad that it was a menu 'cause then you guide me on how the terminology goes, and then you give me exactly what I ask for rather than me trying to tell you what I'm asking." P-005, parent of infant without NBS experience |
|  | “It's nice that there are some questions set up here for me that will make me be able to put together in my head what I really want to know.” P-007, parent of infant without NBS experience |
